# Facile construction of a novel NiFe_2_O_4_@P-doped g-C_3_N_4_ nanocomposite with enhanced visible-light-driven photocatalytic activity

**DOI:** 10.1039/c9na00018f

**Published:** 2019-03-20

**Authors:** Priti Mishra, Arjun Behera, Debasmita Kandi, Kulamani Parida

**Affiliations:** Centre for Nano Science and Nano Technology, Siksha ‘O’ Anusandhan (Deemed to be University) Bhubaneswar-751030 Odisha India kulamaniparida@soa.ac.in

## Abstract

Construction of a Z-scheme-based photocatalyst, *i.e.*, NiFe_2_O_4_@P–g-C_3_N_4_ nanocomposite, was successfully fabricated by coupling phosphorus-doped g-C_3_N_4_ with spinel structure NiFe_2_O_4_. The structural, morphological, and spectroscopic data of the as-synthesized photocatalyst was successfully characterized through XRD, FTIR, SEM, TEM, UV-Vis DRS, PL, and XPS techniques. It was found that NiFe_2_O_4_@P–g-C_3_N_4_ had an increased light-absorption capacity, high exciton separation, low photogenerated electron–hole recombination, and showed better photocatalytic activity toward phenol oxidation and hydrogen energy production than the neat materials. Photocatalytic phenol oxidation by 20 wt% NFO@P–CN was also superior and could achieve a 96% conversion, which was 2 and 3 times higher than that by P–CN and NFO, respectively. The 20 wt% NFO@P–CN showed excellent photostability and was able to evolve 904 μmol h^−1^ H_2_ under visible-light irradiation. The enhanced photocatalytic activity of NiFe_2_O_4_@P–g-C_3_N_4_ was in good agreement with the photocurrent results. The synergistic effect between P–CN and NFO could accelerate photogenerated charge separation and, moreover, the distinctive magnetism of NiFe_2_O_4_@P–g-C_3_N_4_ aided the collection and recycling of the photocatalyst.

## Abstract 2

Fabrication of a novel NFO@P–CN nanocomposite for the photocatalytic degradation of phenol and evolution of H_2_ through a Z-scheme mechanism.

## Graphical abstract

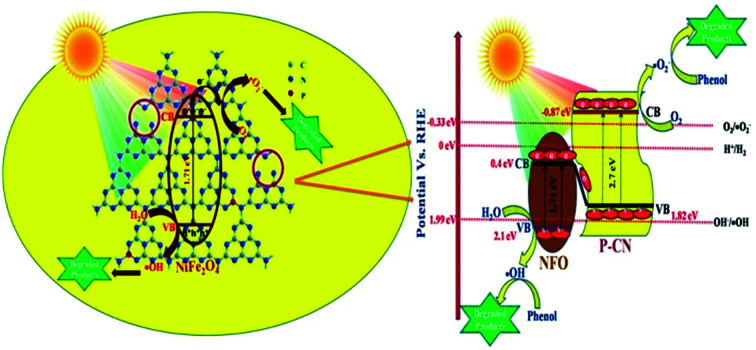

## 1. Introduction

The increasing challenges related to the global clean energy requirement for a progressive world have triggered the research and development of clean energy sources. Due to rapid industrialization and population growth, it is anticipated that by 2050 the global energy requirement will be two times its current supply. During the last few decades photocatalytic technology, as a green sustainable avenue, has attracted extensive attention for the remediation of environmental pollutants and for obtaining chemical energy from solar energy. Semiconductor-based photocatalysts (*e.g.* TiO_2_, ZnO, CdS, Ag_3_VO_4_, ZrO_2_, Ag_2_WO_4_, NiS, and WO_3_) have attracted huge attention and are currently used for several applications in the development of clean energy sources.^1–10^ However, their narrow sunlight absorption range as well as rapid electron–hole recombination limit their practical applications. Therefore, there are challenges still to be overcome for the development of a photocatalyst material with high visible-light absorption, such as how to hinder rapid electron–hole recombination and improve the photocatalytic performances. A number of approaches to develop novel semiconductors are becoming popular, such as the earlier well-coordinated applications of two or more semiconductors through band gap engineering, the construction of nanocomposites, physical property tuning, doping, and making suitable active sites accessible. Recently, composites with a Z-scheme-based system (ZBS) have been developed that have a broader redox range, resulting in stronger oxidizing and reducing powers.^11^ In comparison to a single component system, ZBS photocatalysts represent an excellent approach as they have more positive VB and more negative CB potentials, which independently can give oxidative holes and reducible electrons. As a consequence, this can effectively enhance the photocatalytic performance. Furthermore, it has also been well observed that a direct ZBS photocatalytic system having two components can exhibit better photoactivity, economic feasibility, high stability, and has more promising practical applications than a mediator-assisted ternary Z scheme system.^11,12^ In comparison to heterojunction-type systems, ZBS has shown prospective performance in solar water-splitting as it can easily overcome the thermodynamic energy barrier for solar water-splitting. Also, the complete mineralization of non-selective pollutants can be widely done using this heterogeneous photocatalyst.^13^

Therefore, magnetic semiconductor photocatalysts, such as MFe_2_O_4_ (M = Fe, Ni, Cu, Co, Zn), are in demand for their easy recycling, and consequently, they can be used to check the generation of secondary pollutants. Also, such catalysts show high chemical and structural stability, a good magnetic nature, a narrow band gap, and are visible-light active with potential electric performance.^14^ The higher conductivity of electric charges of MFe_2_O_4_ is because of a hopping process among metal ions of variable oxidation states at O-sites, which is helpful for shifting the charge carriers.^15^ Further, ferrite MFe_2_O_4_, (M = Ni, Zn, and Co) with a spinel structure shows excellent performance toward the degradation of organic pollutants as well as to the evolution of hydrogen by water splitting. The well-known, magnetic semiconductor NiFe_2_O_4_ (NFO) (*E*_g_ = ∼1.7 eV) is unique and in demand because of its wide visible-light-absorption property and admirable photocorrosion and chemical stability. NiFe_2_O_4_ having the chemical formula AB_2_O_4_ is a type of inverse spinel structure, where Ni^2+^ and Fe^3+^ reside in equal number on octahedral sites and are balanced through Fe^3+^ on tetrahedral sites. NFO has a band gap energy of ∼1.7 eV—albeit this is debated despite existing computational results and experimental data.^16^ As per the findings, the presence of Fe^2+^ is attributed to n-type behavior in this catalyst, while the conductivity arises due to the hopping of electrons from Fe^2+^ to Fe^3+^; whereas Ni^3+^ is responsible for the p-type behavior and the conductivity is mostly due to the shifting of holes from Ni^3+^ to Ni^2+^.^17^ Unfortunately, pure NFO exhibits a poor photocatalytic response due to the quick recombination of photoelectron–hole pairs. So, the development of NFO-based photocatalysts is one of the hot and emerging topics in the field of photocatalysis. Therefore, researchers have adopted various effective and successful techniques, such as combining with other semiconductors or co-doping with nonmetals, noble-metal, or polymeric compounds, to improve the photocatalytic performance of NFO-based photocatalysts. Polymeric compounds, like graphitic carbon nitride (g-C_3_N_4_), represent a multipurpose photocatalyst due to their simplistic preparation procedure from inexpensive precursors, suitable band gap, nontoxicity, and high chemical and thermal stability.^18^ Moreover, their proper band gap energy (2.7 eV) as well as the ring structure of tri-s-triazine and greater condensation of this metal-free polymeric semiconductor suggest they have good prospectives as visible-light catalysts. They offer a number of unique advantages, like H_2_ evolution by water splitting, and the ability to degrade organic pollutants from water systems and to eliminate NO from air, due to the CN having a narrow band gap and extended π–π conjugation obtained from its lone pair of electrons. Despite these advantages, inadequate visible-light absorption, a quick recombination of the photoinduced electron/hole (e^−^/h^+^) pairs, their nonmagnetic nature, low electrical conductivity, and imperfect textural properties obstruct the extensive utilization of such compounds in photocatalytic applications.^19^ Recently, the nonmetal modification of CN has proven to be a pivotal approach toward photocatalysis. Nonmetals are suitable for preparing metal-free CN and thus problems due to the variation in oxidation states of doped metal ions during thermal changes can be solved. Again the superior electronegativity and higher ionization energies of nonmetals help them to gain electrons easily, hence making them suitable for covalent bonding. Significant achievements have been gained by phosphorus doping and by the incorporation of phosphorus into the CN matrix, where it helps to narrow the band gap, leading to a noticeable change of color from light yellow (CN) to orange yellow (P–CN) as well as causing a downshift of the VB and the CB positions.^20^

Moreover, the electron-transfer capability and the conductivity can be enhanced by delocalization of the lone electron pairs to the π-conjugated tri-s-triazine of P-doped CN. Phosphorus doping in P–CN improves mass transfer within the reactants and product molecules as well as enhances the specific surface area due to the high porosity, admirable trapping of light, and excellent charge-transfer-induced separation for outstanding hydrogen evolution under visible light. The charge-transfer span from the bulk to the interface is decreased due to the reduced width and the well-developed porosity.^13^ Inverse spinel structure NFO with a P–CN photocatalyst thus represents a small step toward satisfying the environmentally clean energy requirements.^21^

Initially NFO was prepared by a sol–gel method and P–CN prepared by calcination. We here focused on fabricating P–CN-based heterojunction nanocomposites combined with spinel NFO. Taking different mass fractions of NFO, the optimal binary NFO@P–CN was fabricated using a calcination method. Characterizations, including XRD, SEM, HRTEM, and FTIR, were carried out to study the morphology, textural property, particle size allocation, and for verification of the functional groups. Also the optimal binary NFO@P–CN hybrid revealed appreciable optical and electrical properties, and good recycling, stability, and durability when subjected to UV-DRS analysis, PL studies, photoelectrochemical studies, and reusability tests. Further NFO@P–CN was proven to be a promising photocatalyst due to its superior photodegradation performance. It is noticeable that the NFO-doping amount influenced the nanostructure and photocatalytic activity of the NFO@P–CN samples. With respect to pure NFO and P–CN, the 20 wt% NFO@P–CN composite exhibited exceptional photocatalytic performance and greater stability after three runs performed for degrading phenol under solar-light irradiation. Thus, after doping phosphorus in g-CN and pairing with NFO in the Z-scheme-based NFO@P–CN, activity augmentation occurred mainly due to the synergetic effect between P–CN and NFO, resulting in a better absorption of visible light and a greater photoinduced electron–hole pair separation efficiency. Moreover, interest is being shown in this work as no work on phenol degradation or photocatalytic hydrogen evolution by the NFO@P–CN photocatalyst has been reported previously.

## 2. Experimental section

### 2.1 Materials and reagents

Fe(NO_3_)_3_·9H_2_O (99.9%), Ni(NO_3_)_2_·6H_2_O (99.9%), melamine (99.9%), and citric acid (C_6_H_8_O_7_) (99.9%) were obtained from Sigma-Aldrich (analytical grade) and used as-obtained without any additional purification. Deionized water was obtained from a double-distillation unit for preparation of all the aqueous solutions throughout the experiments.

### 2.2 Synthesis protocol

#### 2.2.1 Synthesis of neat NFO

Pure NFO was synthesized by adopting the sol–gel method. In a typical synthesis process, 1 mol of Ni(NO_3_)_2_·6H_2_O and 2 mol of Fe(NO_3_)_3_·9H_2_O were dissolved in 100 mL deionized water (solution A), while 3 mole of citric acid was dissolved in 100 mL deionized water (solution B), where the metal (Ni^2+^ + Fe^3+^)/citric acid molar ratio for the respective solutions was 1.

**Scheme 1:**
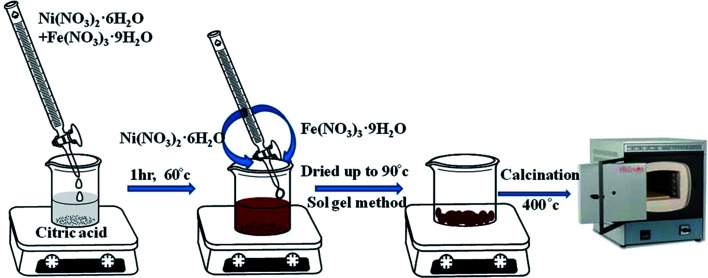
Synthesis of NiFe_2_O_4_ by the sol–gel method.

Then, under vigorous magnetic stirring, solution A was added dropwise into solution B. The reaction was carried out for 1 h at 60 °C and the mixed aqueous solution was then dried at 90 °C. The dried powder obtained was calcined at 400 °C for 4 h with a 10 °C min^−1^ temperature increase, as shown in Scheme 1. The calcination reactions are given below.^15^

1 Ni(NO_3_)_2_ + 6H_2_O → NiO + NO_2_ + NO + 6H_2_O + O_2_

2 2Fe(NO_3_)_3_ → Fe_2_O_3_ + 3NO + 3NO_2_ + 3O_2_

3 2C_6_H_8_O_7_ + 9O_2_ → 12CO_2_ + 8H_2_O

4 NiO + Fe_2_O_3_ → NiFe_2_O_4_ (NFO)

The exothermic reaction between nitrates and citric acids liberates enormous heat. This helps in decomposing the direct chains in citrates and allows contact with the homogenously distributed adjacent atoms, resulting in the formation of a crystal lattice. Citric acid/metal (Ni^2+^ + Fe^3+^) in the ratio of 1 : 1 burns the NO_3_^−^ ions and the carboxyl groups completely. The high heat generated from the exothermic reaction of nitrates and citric acid gives rise to the higher degree of compositional homogeneity.

The properties of NFO are very susceptible to the synthesis conditions. By changing the oxidation state of the nickel or iron cations present in the octahedral sites of the inverse spinel structure, NFO can be either oxidized or reduced. It has already been reported that by varying the synthesis conditions, NFO can have either p-type or n-type conductivity.^17^

#### 2.2.2 Synthesis of neat g-CN

Melamine (Sigma-Aldrich, analytical grade) was used to synthesize neat g-CN by calcination using a temperature rise of 10 °C min^−1^ directly at 550 °C for 4 h.^13^

#### 2.2.3 Synthesis of doped P–CN

**Scheme 2:**
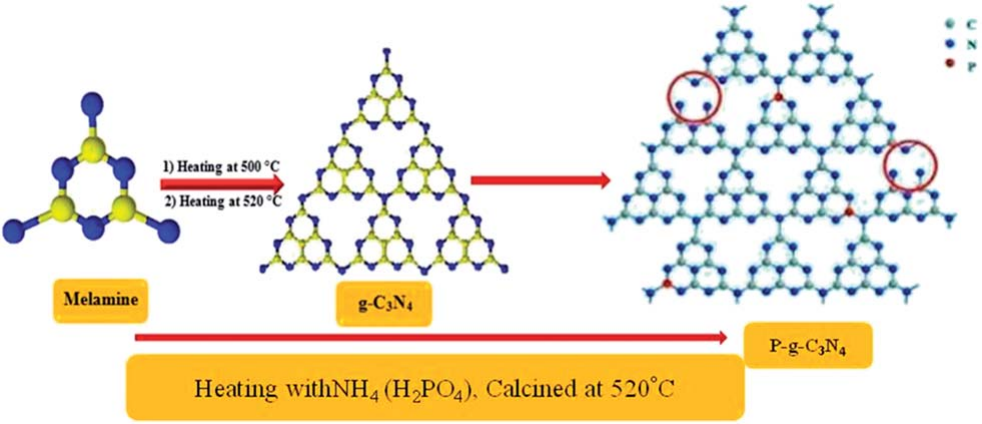
Synthesis of P–g-C_3_N_4_ by the calcination method.

Phosphorus-doped g-CN was synthesized by slowly adding 2 g of NH_4_ (H_2_PO_4_) to an aqueous solution of 10 g of melamine. Then at room temperature (*ca.* 25 °C), the solution was stirred for 1 h and finally completely dried by heating at 50 °C. Further, the powdered sample was placed in an enclosed ceramic crucible and calcined at up to 520 °C (at 3 °C min^−1^) and then left for 2 h till properly cooled down to room temperature. Thus, P–CN was prepared as shown in Scheme 2.^22^

#### 2.2.4 Fabrication of the NFO and P–CN nanocomposites (NFO@PCN)

NFO@P–CN was prepared by the sol–gel route in order to improve the photocatalytic performance for the degradation of phenol using solar light. Nanocomposites were prepared by taking different mass fractions of NFO@P–CN, through the calcination method. Here, 0.1 g P–CN and different mass amounts of NFO were mixed homogeneously by grinding in an agate mortar for at least 30 min, followed by calcination at 400 °C with a heating rate of 10 °C min^−1^ for 4 h. The mass fraction of NFO in *x* NFO/g-C_3_N_4_ was varied as *x* = 10, 20, 30, 40 and 50 wt%.^15^

### 2.3 Formation mechanism

Due to the good harmony between P–CN and Ni^2+^ ions, *in situ* calcination favors the interaction between P–CN and NFO. The presence of –NH_2_ groups of CN gives rise to an electrostatic attraction, which helps phosphorus get attached on the facades of CN. Electronically deficient Ni^2+^ and Fe^3+^ are attracted easily by CN, as it is considered a very good Lewis base owing to the presence of a lone pair of electrons on the N-atoms of CN. Further, the magnetic NFO nanoparticles were spread out on the P–CN sheets and could not only enhance the spreading capacity of the layered composite but could also enhance photocatalytic reactions by furnishing a greater number of reaction sites. The interaction between P–CN and NFO was through an electrostatic synergistic effect and physisorption. Notably during the formation P–CN and NFO molecules, they formed a granules-embedded-layered-type nanocomposite with inbuilt benefits of being stable, effortlessly separable, and superior handling properties. Additionally, the development of the redox photocatalyst was environmentally friendly, economical, and allows redox modification between inconsistent oxidation states.^23^

### 2.4 Methods of characterization

The crystallinity, phase composition, and crystal structure of the as-synthesized materials were examined using a Rigaku Miniflex Advance X-ray diffractometer (set at 30 kV and 15 mA) using Cu Kα radiation (*λ* = 1.54056 Å). Taking a 2*θ* angle ranging from 10° to 80° with a scanning rate of 8° min^−1^ and a step size of 0.01°, the X-ray diffraction arrangements were carried out. Scanning electron microscopy (SEM, Hitachi S–3400N) was used to measure the surface framework and the composition of the synthesized samples and a ZEISS SUPRA 55 instrument was used for FESEM analysis. A 3 mm diameter fine-mesh copper grid coated with a carbon film was used to support the powder particles. A thin film of gold was used to sputter the sample prior to the analyses. To investigate the morphology interaction between the NFO and P–CN and the microstructure of the prepared catalysts, a TEM-JEOL-2010 200 kV instrument was used to get the transition electron microscopy (TEM) spectra at different scales. High-resolution transmission electron microscopy was used for the lattice fringe measurements. The chemical composition and vibrational modes of the NFCN samples were analyzed at the frequency range of 2500–400 cm^−1^ with the help of a Perkin-Elmer RXI FT-IR spectrophotometer having a resolution of 4 cm^−1^. KBr was used as the reference diluent. UV-Vis spectra were collected using a UV-visible spectrophotometer (JASCO-750) and the Kubelka–Munk method was used to transform from the reflection to absorbance with BaSO_4_ as the reflectance reference. X-ray photoelectron spectroscopy (XPS) was executed with a monochromatic X-ray source (Mg Kα X-ray) using a VG Microtech Multilab ESCA 3000 surface analyzer. Photoluminescence (PL) spectra were investigated with a fluorescence spectrophotometer (JASCOFP-8300) at room temperature, with a xenon lamp used as an excitation source having an excitation wavelength of 330 nm.

#### 2.4.1 Photoelectrochemical measurements

The photoelectro chemical (PEC) response of the synthesized nanocomposite was analyzed by Mott–Schottky (MS), cyclic voltammetry (CV), electrochemical impedance spectroscopy (EIS), and linear sweep voltammetry (LSV) performed under the required conditions. Photocurrent studies and measurements were carried out on an Auto LAB electrochemical analyzer (Ivium potentiostat) using a standard three-electrode system. Further, the prepared electrode, a Pt wire, and Ag/AgCl were taken as the working electrode, counter electrode, and reference electrode, respectively. A Na_2_SO_4_ (1.0 M) aqueous solution of pH 6.7 was chosen as the electrolyte for the study. A 300 W Xe lamp filtered through an UV cutoff filter (*λ* = 400 nm) served as the source of light. Photoelectrochemical (PEC) measurements were performed in a Pyrex electrochemical setup and the working electrode was obtained by an electrophoretic deposition processes with FTOs, which included 2 NFCN samples (deposited on FTO). The electrodes were obtained by electrophoretic deposition using iodine (0.02 mg) solution in acetone (30 mL) with the photocatalyst powder (0.02 mg) for the photoelectrochemical measurements. A 60 V bias was used among the two parallel FTO (fluorine-doped tin electrodes) separated by 10–15 mm dipped in the solution for 3 min under potentiostat control. The FTOs were dried, with a coated area of 1–3 cm^2^. Using an AC voltage of 10 mV amplitude, EIS was performed keeping within the frequency array of 0.1 Hz to 100 kHz. Pt wire and Ag/AgCl were taken as the counter and the reference electrodes, respectively. In the dark condition at 1500 Hz, the Mott–Schottky plot of the photocatalyst was noted. Finally, by performing a potential sweep from to −0.2 to 0 and 0 to 1.5 V the polarization curves (LSV) were produced.

### 2.5 Photocatalytic experiments

#### 2.5.1 Photocatalytic hydrogen evolution measurements

The hydrogen production reactions were analyzed in a 100 mL sealed Pyrex quartz reactor round-bottom flask connected with an enclosed gas distribution as well as evacuation system A medium-pressure 150 W xenon arc lamp (*λ* > 420 nm) was used as a light source to trigger the reaction *via* a 1 M NaNO_2_ solution as the UV cutoff filter was placed 20 cm away from the photoreactor. The average light energy density was measured to be 120 mW cm^−2^. In the typical photocatalytic water-splitting experiment, 20 mg of the target powdered catalyst in 20 mL aqueous solution having 20% of triethanolamine (TEOA) solution was taken. Triethanolamine (TEOA) was also used to optimize the conditions for H_2_ evolution. To maintain uniformity, the suspension was constantly stirred throughout the reaction and prior to irradiation; it was finally deaerated and purged with N_2_ gas for 20 min for complete removal of CO_2_ and O_2_ diffused in water. The volume of hydrogen gas evolved was collected by the downward displacement of water. By performing reusability tests, the quantity of hydrogen furnished by the photocatalyst with time and stability were evaluated.

#### 2.5.2 Experiment for phenol oxidation

Phenol, a distinctive toxic aromatic compound having low degradability by conventional decay processes, was selected for measuring the photodegradation efficiency of the prepared photocatalyst at different pH (2, 3, 4, 6, 8). All the experiments, including the direct photolysis of phenol and the adsorption of phenol on catalysts in the dark, were performed in the day time within the period 10:00 am to 12:00 noon. For substrate-model pollutant equilibration, the dispersed photocatalyst with the pollutants was magnetically stirred for 30 min at room temperature in the dark. Then, 0.02 g of the as-prepared catalyst at pH = 6 was placed in a magnetically stirred conical flask carrying 20 mL of 20 ppm phenol. In order to accomplish adsorption–desorption equilibrium, the aqueous suspensions were subjected to 30 min stirring in the dark, followed by degradation activity using direct sun light for 2 h. Among all the tested pH (2, 3, 4, 6, 8), pH = 6, was found to be optimum for phenol oxidation. Then, the aqueous catalyst aliquots were immediately centrifuged to get rid of the residual catalyst and analyzed using an UV-Vis spectrophotometer to find out the oxidation of phenol by the catalysts. Phenol concentrations were calculated calorimetrically at 510 nm after 1 h by using 4-aminoantipyrine as a color indicator.

## 3. Results and discussion

### 3.1 Study of the crystal structure, composition, and morphology of the as-prepared samples

#### 3.1.1 Powdered X-ray diffraction (XRD) studies

**Fig. 1:**
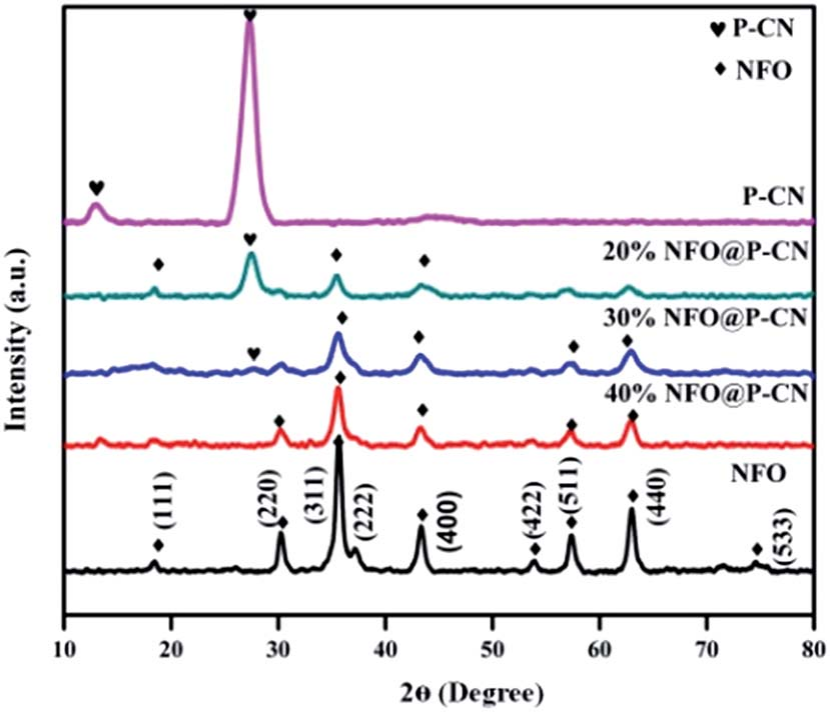
XRD spectra of NFO, P–CN, and the NFO@P–CN composite.

The structural identification and crystal phases of the neat NFO, P–CN, and nanocomposites *x* NFO@P–CN (*x* = 10, 20, 30, 40, and 50 wt%) were analyzed by XRD. The peaks at 18.4°, 30.3°, 35.7°, 37.3°, 43.4°, 53.8°, 57.4°, 62.9°, and 74.6° are attributed to the (111), (220), (311), (222), (400), (422), (511), (440), and (533) planes of NFO, as shown in Fig. 1 (JCPDS no. 10-0325).^16^ Additionally, the doped P–CN exhibited two diffraction peaks at 13.1° and 27.5° (*d* = 0.322 nm) referring to the (100) and (002) crystal planes, which correspond to the typical crystal stage of the inter-layer stacking and aromatic network.^24^ Hence, it was found that the above two peaks were similar to pristine CN. But more interestingly, compared with CN, a minor shift toward a greater 2*θ* value (27.5°) was observed for P–CN, which is well consistent with previous reports.^25^

After P doping, the (002) peak of CN was decreased, indicating an enhanced interlayer distance from 3.22 A° to 3.24 A°, which might be ascribed to the increase in radius of the doped P atom (100 pm) with respect to the supplant C atom (70 pm).^26^ Also, the pattern revealed that with the increase in NFO content from 10–50 wt%, a sharp decrease in intensity was observed for P–CN. This might be due to the massive amount of heat generated by the decomposition of nitrate, which could possibly damage the crystalline structure of P–CN. On the other hand, with the increasing NFO content in the composite, there is a gradual increase in the diffraction intensity of the peak at 35.5°. However, apparently no peak shift occurred for NFO, indicating that P–CN has no influence on the crystal arrangement during the calcination route in the nanocomposite synthesis. Furthermore, these results suggest that the NFO photocatalysts stick to the CN surface but are not incorporated into the lattice of the CN.

There is a possibility for the chemical bonding of P atoms in P–CN with the C and N neighbors, which would endow a planar coordination in the structure. Thus the crystal growth of CN may be hindered by phosphorus doping, which decreases the band gap and enhances the photogenerated electron–hole separation. P atoms prefer to be located in the bay-carbon and substitutional corner carbon sites, whereas the P–N bonds are produced by the interstitial doping of P into the CN lattice, as reported by Zhou *et al.* Therefore, both interstitial and substitutional doping are strongly influenced by the phosphorus precursors. Hence a good integration of P–CN with NFO was observed from the as-obtained XRD pattern.^27^

#### 3.1.2 FTIR

**Fig. 2:**
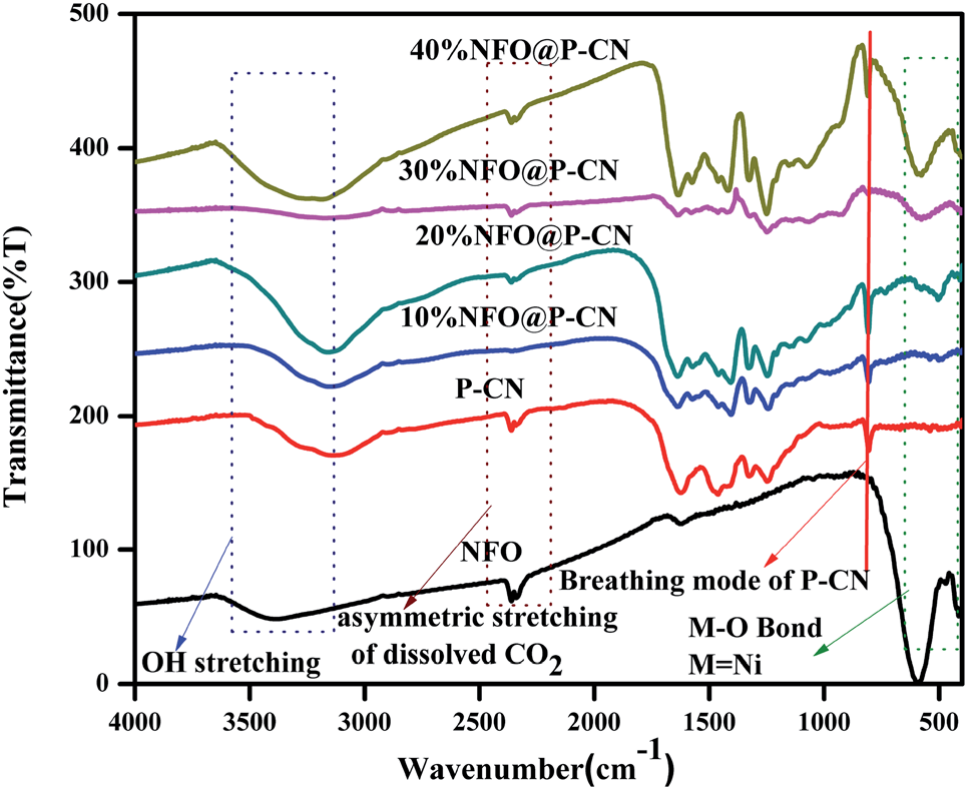
FTIR spectra of the NFO, P–CN, and 10–50 wt% NFO@P–CN composites.

The chemical composition and vibrational modes of NFO@P–CN samples were analyzed by FTIR spectroscopy, as shown in Fig. 2. The formation of P–CN as well as CN was well confirmed through the FT-IR spectra. The spectrum for P–CN typically well resembles that for to CN. From the P–CN spectrum, the broad peak in the region 3100–3500 cm^−1^ (centred at 3400 cm^−1^) can be ascribed to a stretching vibration of uncondensed –NH and also represents the surface-adsorbed water molecules. Again the spectrum reveals the typical stretching band around 1200–1650 cm^−1^ and a series of peaks at around 1250, 1325, 1410, 1560, and 1632 cm^−1^ assigned to the bridging C–NH–C or trigonal C–N(–C)–C stretching modes of CN heterocycles. A weak peak located at 950 cm^−1^ can be assigned to P–N stretching vibration and it seems to overlap with aromatic C–N vibration. Moreover, an intense peak at around 807 cm^−1^ can be typically assigned to the characteristic bending vibration of a s-triazine ring.^22^ For NFO, the peak at around 1650 cm^−1^ was ascribed to the bending vibration of OH. A weak absorption peak at 417 cm^−1^ and a strong peak at 608 cm^−1^ were assigned to Fe–O bonds in the tetrahedral and stretching vibrations of metal–O bonds within the octahedral positions.^28^ All the characteristics peaks for P–CN as well as NFO were well maintained in the *x*-NFO@P–CN spectrum, which confirmed the presence of NFO and P–CN.

#### 3.1.3 SEM

**Fig. 3:**
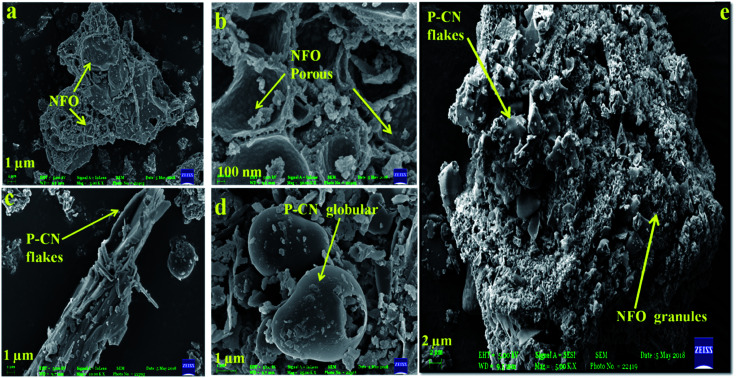
(a, b) SEM image and enlarged view of NFO, (c, d) flake-like and globular structure of P–CN, and (e) 20 wt% NFO@P–CN hybrid nanomaterial.

The SEM images of the P–CN-based composites are shown in Fig. 3(a–e). The P–CN, NFO, and NFO@P–CN nanomaterials revealed irregular microstructures of granules and sheets having a thickness range of 20–30 nm (Fig. 3a–d).^20^

It was found that pure NFO exhibited an unstructured granule-like nature (Fig. 3a and b), whereas pure P–CN showed a flake-like structure and big globular agglomerates with a smooth surface (Fig. 3c).^29^ This was attributed to the fact that the variation in nanostructure of P–CN is influenced by the synthesis route utilized. As shown in Fig. 3c, the morphology of P–CN appeared to be smooth and maintained the original special layered morphology of CN, indicating the layer structure is not altered after phosphorus doping. The 20 wt% NFO@P–CN nanocomposite in Fig. 3e reveals the presence of both P–CN as well as NFO particles. The SEM image in Fig. 3e unveils that the surface of P–CN particles are covered by irregular, agglomerated, porous NFO particles. Also, due to the flake-like structure of P–CN, the thickness is reduced, which thus increases the charge-transfer mobility. Hence, the thin nanosheets are quite promising for enhancing the photoredox reactions during photocatalytic activity.^25^ This type of morphology facilitates the formation of well-managed composites as well as helps electron channelizing, thus making it suitable for photocatalytic activity applications.^20^

#### 3.1.4 TEM

**Fig. 4:**
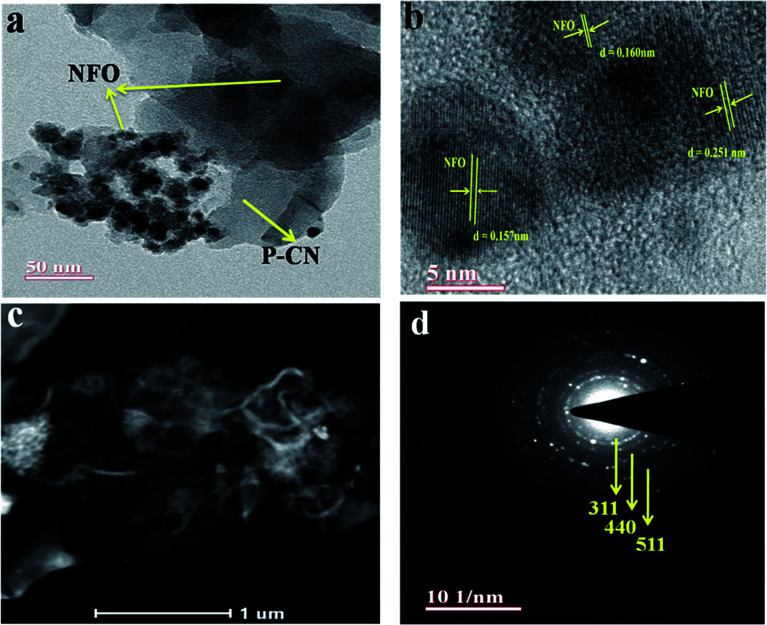
(a) TEM image of the NFO decorated over the P–CN sheets. (b) The HRTEM image showing the lattice fringes of the different planes of 20 wt% NFO@PCN nanocomposite. (c) HAADF image of 20 wt% NFO@PCN. (d) SAED pattern of 20 wt% NFO@PCN.

The well-defined composite formation between the P–CN and NFO phases in the 20 wt% NFO@P–CN hybrid nanomaterial was explored using TEM. As shown in Fig. 4a, the unstructured granule-like nanoscale particles referred to pure NFO. NFO is closely coupled with P–CN, as shown in Fig. 4a–c. In Fig. 4a, it can be clearly seen that NFO has two dissimilar types of grain structures: large polygon granules (25–50 nm) and nanosized particles (about 5 nm).^16^ So the crystallite size distraction of NFO could be suitably bimodal. Fig. 4b presents a distinctive HRTEM image of NFO with lattice planes of 0.251, 0.160, and 0.157 nm, consistent with the mean value of the basal spacing of the (311), (440), and (511) planes, respectively.^30^

Also, these are in tune with the (311), (440), and (511) planes computed from the XRD results (*d* = 0.2514 nm and *d* = 0.322 nm).^16^ The HAADF pattern in Fig. 4c illustrates that NFO porous granules and nanoparticles are adhered to the P–CN flake and that the dispersion of NFO nanoparticles is also noticeable, which would be beneficial to promote the separation efficiency of photoinduced carriers. Furthermore, the crystallinity of the nanocomposites was confirmed by the selected area electron diffraction (SAED) pattern (Fig. 4d).

#### 3.1.5 XPS

**Fig. 5:**
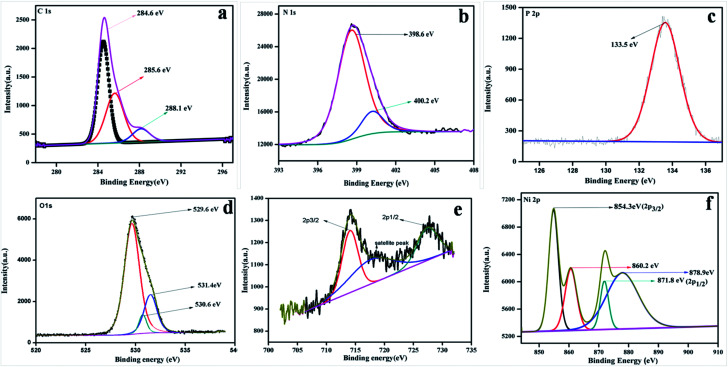
XPS spectra for 20 wt% NFO/PCN in the regions of: (a) C 1s, (b) N 1s, (c) P 2p, (d) O 1s, (e) Fe 2p and Ni 2p.

To study the surface chemical composition and the oxidation state of the as-prepared photocatalyst, XPS was carried out. Fig. 5a–e represent the high-resolution spectra of C 1s, N 1s, O 1s, Fe 2p, and Ni 2p for the 20% NFO@PCN photocatalyst. In Fig. 5a, the peaks at 284.6 eV and 288.1 eV are attributed to pure graphitic carbon (C–C) and to the characteristics peak for N–C=N co-ordination in P–CN, respectively.^25^ Another peak at 285.6 eV is the characteristic peak for C–O.^31^Fig. 5b depicts the XPS spectra for N 1s, which has been deconvoluted into two peaks at 398.6 and 400.2 eV. The peak at 398 eV can be ascribed to sp^2^ hybridised nitrogen in C=N–C, while another peak at 400.2 eV can be for tertiary N–(C) 3 groups.^16^ The presence of P integrated into the CN structure was confirmed from the P 2p binding energy peaks at *ca.* 133.5 eV (133.4–133.6 eV), indicating a P–N coordination (Fig. 5c).^22^Fig. 5d reveals the spectrum for O 1s. The peak found at 529.6 eV is due to the presence of O^2−^ ions in the crystal lattice of NFO; whereas peaks at 530.6 eV and 531.2 eV are assigned to chemisorbed oxygen from the surface adsorbed water or hydroxyl (OH^−^)-type oxygen species and oxygen vacancies, respectively. Fig. 5e represents the XPS spectra for Fe 2p and shows three peaks. A satellite signal at about 718 eV is accompanied by the Fe 2p_3/2_ and Fe 2p_1/2_ peaks, which certify the existence of only Fe^3+^.^32^ The peaks at 714.2 and 727.5 eV are ascribed to the binding energy of Fe 2p_3/2_ and Fe 2p_1/2_. The shake-up feature is associated with the main peaks of ionic Fe (Fe^3+^). It is also observed that the XPS spectrum of Ni 2p does not conflict with the earlier reported literature.^33^ Both the peaks at 854.3 and 871.9 eV, as the distinctive binding energy of Ni^2+^, correspond to Ni 2p_3/2_ and Ni 2p_1/2_, as shown in Fig. 5f. Further, two more peaks with 860.8 and 878.9 eV binding energies correspond to the satellite peaks. From the XPS analysis, the presence of P was confirmed as a dopant. Thus, as reported by Hu *et al.* earlier, the bay or corner carbon in the structure may be replaced by the P heteroatom, thereby forming P–N bonding in the doped CN. Again, as prepared from melamine, the surface concentration of phosphorus in P–CN was the lowest, as well as it forming two P–N bonds occupying the interstitial positions (as confirmed from the FTIR results, Fig. 2).^22^

## 4. Electronic band structure and optical properties

### 4.1 UV-DRS

**Fig. 6:**
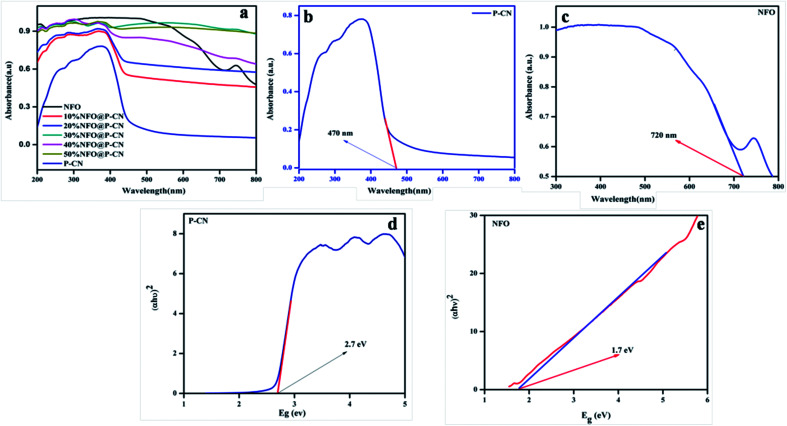
(a, b and c) Absorption spectra of the NFO, P–CN, and 10–50 wt% NFO@P–CN, and (d, e) band gap energy of P–CN, NFO respectively.

UV-Vis diffuse reflection spectroscopy (DRS) was used to study the optical absorption properties of the neat NFO, P–CN, and (10–50) wt% NFO@P–CN in the range of 200–800 nm and the results are depicted in Fig. 6a–c. The spectra indicate that the NFO composite has a wider power full absorption region between 200 and 720 nm and P–CN shows a sharp absorption edge at 450 nm. Upon increasing the percentage of NFO in the NFO@P–CN composites, there is a red-shift observed, hence absorption extends from the visible to near IR region. Thus, the prepared composites are strongly photoresponsive to the solar spectrum. Further the band gap value was estimated by the Kubelka–Munk equation.

5 *αhν* = *k*(*hν* − *E*_g_)^*n*/2^

According to eqn (5), a plot was made by taking the energy *versus* the Kubelka–Munk function.

The function is expressed by *α*, which denotes the absorption coefficient, while *hν* denotes the photon energy, *E*_g_ the band gap, *k* is a constant, and the type of transition gives rise to *n* values. For direct and indirect transition, *n* = 1 and 4, respectively. In Fig. 6d and e, the results indicate that the calculated band gap values for the direct band gap semiconductor NFO is approximately 1.71 eV and that of indirect transition in P–CN is 2.7 eV.^16,28^ The band gap energy of *ca.* 2.7 eV and lone pair of electrons on the N atoms of the triazine/heptazine ring system in P–CN give rise to n–π* transitions, which are responsible for a strong absorption band edge at 450 nm. In the case of NFO, the strong absorption up to 720 nm is due to the transition of Ni_2_^+^–O–Fe_3_^+^ to Ni^+^–O–Fe_4_^+^. The Ni^2+^ ions with the d^8^ configuration in the octahedral field are responsible for the spin-allowed transitions of 3A2g (F) → 3T1g (P) and 3A2g (F) → 3T1g (F), which correspond to the absorption bands in between 300 and 720 nm.^34^ Therefore the introduction of NFO enhances the surface electric charge of P–CN-based composites and thus make them suitable as a visible-light photocatalyst. This may be due to a restraining of the contact barrier and enhancement of the electronic coupling between dissimilar nanocomposites. Hence the UV-Vis DRS outcome for (10–50) wt% NFO@P–CN showed the evolution of more photogenerated charges and an enhancement in light absorption, thus enhancing the photocatalytic activity of the photocatalyst.

### 4.2 Confirmation of the photogenerated electron–hole separation from PL spectral analysis

**Fig. 7:**
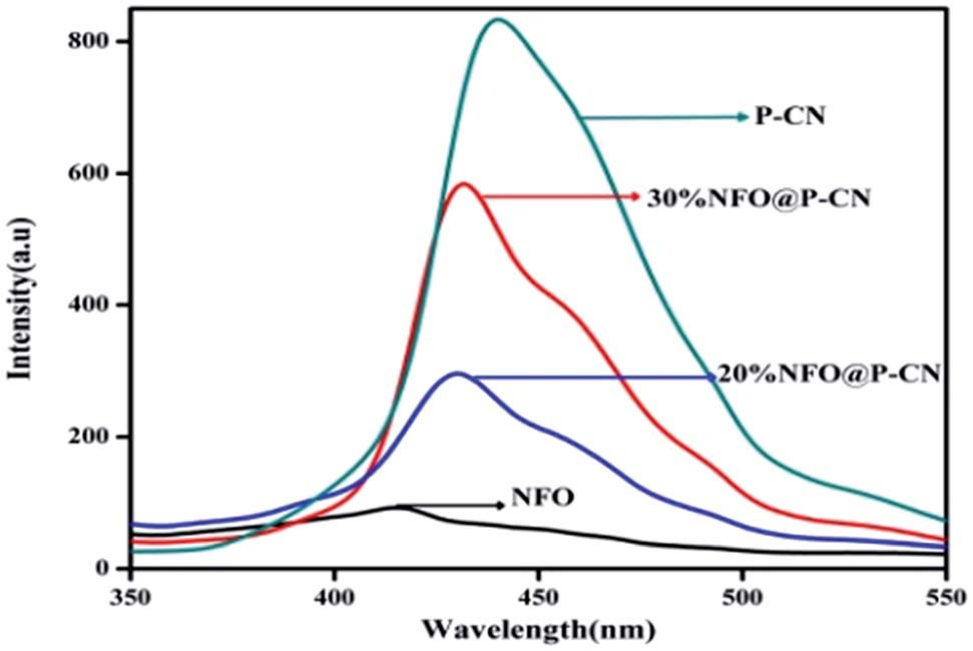
PL spectra of NFO, P–CN, and 20 and 30 wt% NFO@P–CN at an excitation wavelength of *λ*_ex_ = 350 nm.

In semiconductors, the transfer, recombination, and migration of photogenerated charge carriers are related to the photoluminescence (PL) emission spectra. The maximum separation of electron–hole pairs is confirmed by the lower intensity of the PL spectra. We carried out PL analysis to investigate the separation efficiency and charge-carrier transformation of the 10–50 wt% NFO@P–CN nanocomposites and neat samples. In Fig. 7, the characterized PL spectra of the prepared nanocomposites are shown under the excitation wavelength at 350 nm. At around 440 nm, the neat P–CN sample revealed a strong luminescence, corresponding to a near band gap energy of 2.7 eV. This might be due to the n → π* electronic transition arising from the availability of the lone pair of electrons on the N-atom.^24^ Further P doping enhances the dispersal of the contour allocation of the HOMO and LUMO and smoothens the augmentation of carrier flexibility, while the noncoplanar HOMO and LUMO support photogenerated electron–hole pair separation. The interstitial P-doped CN expanded conjugated system might be the reason for this.^24^ All the catalysts exhibited analogous PL spectra, whereas weak peaks for NFO were observed in the range of 380 nm to 500 nm, which might be ascribed to the surface oxygen vacancies and defects of NFO originating from excitonic PL.^31^ After the addition of NFO species, an obvious decrease in the intensity of the PL spectra for P–CN was observed, suggesting that NFO modification efficiently prevented electron–hole pair reunion. Finally from these characterizations, well-supporting results in favor of photoinduced charge migration and separation were obtained for 20 wt% NFO@P–CN.

### 4.3 Photogenerated electron–hole transport from EIS analysis

**Fig. 8:**
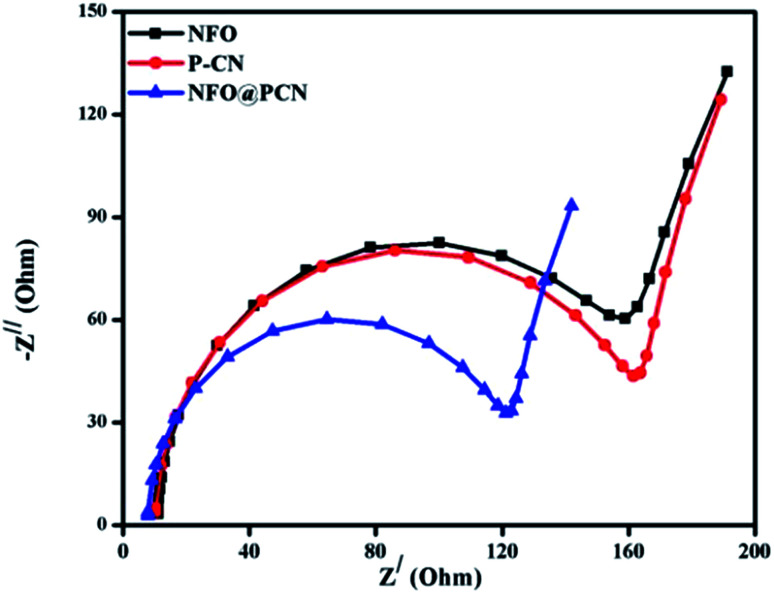
Nyquist plot for the NFO, P–CN, and 20 wt% NFO@P–CN nanocomposites.

The efficiency of the charge-transfer process and charge-carrier separation at the electrodes can be well explained by EIS. In Fig. 8, the EIS spectra of P–CN, NFO, and NFO@P–CN composites are shown by a distinctive Nyquist diagram explaining photogenerated charge separation, charge transfer, the rate of reaction at the surface of electrode, and the resistance given by the photocatalysts under study. It is well known that constant-phase element (CPE) and the charge-transfer resistance (*R*_ct_) at the interface of a composite-electrolyte is contributed by the semicircle part of the EIS plot. The high-frequency semi-circular part corresponds to the conductivity of the catalyst, while the low-frequency straight-line part refers to the Warburg resistance. The consensus is that the smaller the semicircle, the lesser is the charge-transfer resistance at the interface and the higher is the conductance.^35^ Conductivity arises due to the hopping of electrons from Fe^2+^ to Fe^3+^ and by the shifting of holes from Ni^3+^ to Ni^2+^ in NFO and by delocalization of the lone electron pairs to the π-conjugated tri-s-triazine in P–CN.^17^ In the case of the 20 wt% NFO@P–CN composite, a reduced arc radius is clearly indicated by the Nyquist plot (*Z*_img_*vs. Z*_real_), revealing its superior conducting efficiency. From the plot, the charge-transfer resistance of the neat and of the 20 wt% samples was found to be in the order: 20 wt% NFO@P–CN (112.00 Ω) < NFO (150 Ω) < P–CN (152 Ω). In addition, the greater the diffusion or the transfer of ions in the electrolyte, the smaller the straight-line part. Hence with the smallest straight line part, the 20 wt% NFO@P–CN nanocomposite showed the best electrical conductivity as well as the highest electron–hole pair separation, which helps in enhancing the photocatalytic performance.

### 4.4 MS, LSV, and PC measurements by electrochemical study

We gained more insights into the mechanism through performing a Mott–Schottky plot (MS), linear sweep voltammetry (LSV), and testing the charge shifting speed at the electrode/electrolyte interface to understand the mechanistic pathway for the NFO@P–CN nanocomposites.

#### 4.4.1 Mott–Schottky plot

**Fig. 9:**
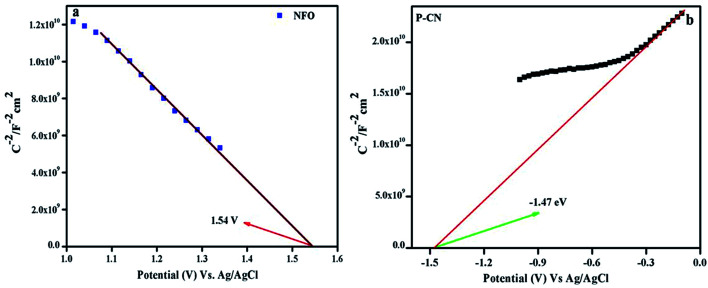
Mott–Schottky plots of (a) NFO and (b) P–CN.

The consequences on the charge carriers and the type of composite material were studied by drawing the MS plots of the NFO and P–CN (Fig. 9a and b). Considering the MS plot as a function of the potential, the existence of a p–n composite indicates the presence of both +ve and –ve charge carriers. The p-type character of NFO was confirmed by its negative slope, whereas the n-type nature of the P–CN electrode was established by its positive slope. As per the finding, Ni^3+^ was responsible for the p-type behavior and the conductivity was mostly due to the shifting of holes from Ni^3+^ to Ni^2+^. The flat band potential (*E*_fb_) for NFO and P–CN was obtained from the intercept of the *x*-axis by the slope during extrapolating the graph to *C*^−2^ = 0. The *E*_fb_ is roughly equal to the VB potential for p-type semiconductors and the CB potential for n-type semiconductors. Using the Nernst equation (eqn (6)), the *E*_fb_ could be altered into a normal hydrogen electrode (NHE).

6 *E*_fb_ (*vs.* NHE) = *E*_fb_ (pH = 0, *vs.* Ag/AgCl) + *E*_AgCl_ + 0.059 × pH

Hence, the *E*_fb_ potential for the p-type was found to be 1.54 eV and for the n-type was −1.47 eV with respect to Ag/AgCl, whereas with respect to NHE these values were 2.1 eV and −0.87 eV, respectively. Again from the UV-DRS measurements, the band gap was found to be 1.7 eV and 2.7 eV for NFO and P–CN, respectively. Thus the VB for P–CN and the CB for NFO were calculated as 1.8 eV and 0.4 eV, respectively. The CB edge of P–CN (−0.87 eV *vs.* NHE) was more negative in contrast to that of p-type NFO (+0.4 eV *vs.* NHE).

#### 4.4.2 Linear sweep voltammetry

The linear sweep voltammogram (LSV) technique was also used to establish the photoelectrochemical activities of the neat NFO, P–CN, and composites, *i.e.*, the 20 wt% NFO@P–CN photoelectrodes. Under visible light irradiation, the charge-separation efficiency of the as-prepared nanocomposites was estimated by measuring the photocurrent density. Initially, Ag/AgCl was used as the reference electrode in 0.1 M Na_2_SO_4_ solution at 25 °C to measure all the potentials, but the ultimate results are given related to NHE.

**Fig. 10:**
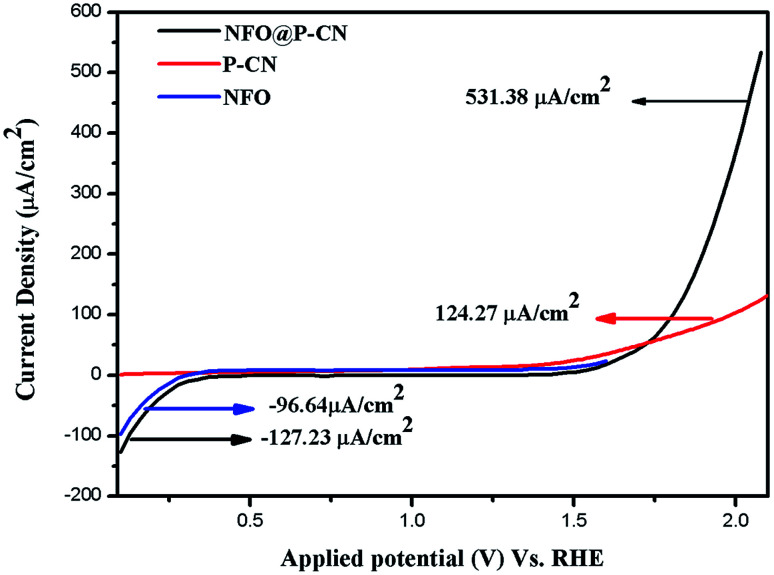
Photocurrent density of the NFO, P–CN, and 20 wt% NFO@P–CN nanocomposite.

All the activities for the as-prepared NFO, P–CN, and 20 wt% NFO@P–CN photoelectrodes were performed both under light and in the dark using a scan rate of 0.05 mV s^−1^. The charge-separation ability was studied using a photocurrent and calculated by removing the dark current part from the overall current to take off the effect of dark currents. Polarization arcs were here used to find out the photocurrent activities of all the prepared photocatalysts. An insignificant response was experienced for the observed potential of all the neat materials NFO and P–CN, confirming their low electrocatalytic achievement. Under light irradiation, neat NFO showed photocurrent both in the cathodic and anodic directions, but a significant increase was found toward the cathodic direction with respect to the applied bias, confirming it as a p-type semiconductor; whereas P–CN proved to be an n-type semiconductor under light conditions. A cathodic current density of −96.64 μA cm^−2^ was obtained due to the occurrence of the p-type NFO, finally ending in a saturated value. The 20 wt% NFO@P–CN nanocomposite exhibited an anodic current density of +531.38 μA cm^−2^ and a cathodic current density of −127.43 μA cm^−2^ in the range of the applied potential. Hence with the relevant positive potential, the anodic current density was increased, thus enhancing the distinctive n-type behavior, as shown in Fig. 10. Both the anodic and cathodic photocurrents confirmed the oxidation and reduction responses of the 20 wt% NFO@P–CN, recognizing consecutive oxidation–reduction processes. At a potential of 1.5 V against Ag/AgCl, a superior anodic photocurrent density was obtained with respect to the cathodic region. This suggested that by combining NFO with P–CN, there was an enhancement in the oxidizing property. Under visible light irradiation, the enhanced current density as well as the photocatalytic activity might be attributed to the harmonious effect of phosphorus in P–CN, enhanced electron shuttling followed by the rapid separation of photoinduced electron–hole pairs, and the low resistance given by the composite.^32^

## 5. Photocatalytic activity

To evaluate the photocatalytic performance of the as-synthesized photocatalyst, phenol degradation as well as H_2_ evolution were performed using visible light irradiation.

### 5.1 Photocatalytic oxidation of phenol

The photocatalytic activity of the neat and NFO@P–CN nanocomposite samples was determined by their ability to degrade 20 ppm phenol as a specimen pollutant of the ecosystem. The photodegradation procedure involved a 30 min adsorption of phenol on the catalyst surface, which resulted in a very nominal change of phenol concentration. In addition to this, in order to investigate the type of reaction, the degradation procedure was also performed in the absence of the photocatalyst. The obtained results indicated the involvement of a photocatalysis process rather than photolysis. Initially, at different pH, the activity was also studied. At pH < 7, the oxidation percentage was increased, whereas at pH > 7 the degradation was found to be decreased. So, as per the observed results, pH 6 was taken as the optimum pH and studied for the degradation of phenol under solar-light irradiation.

**Fig. 11:**
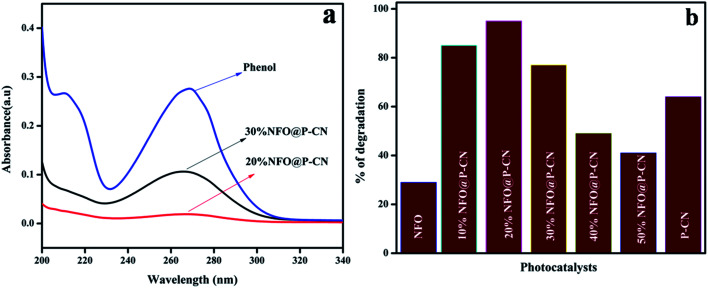
(a) Adsorption spectra of the prepared catalysts in phenol degradation, (b) comparison of the percentage of degradation of phenol by the NFO, P–CN, and 10–50 wt% NFO@P–CN nanocomposite.

Fig. 11a shows the spectral presentation of phenol degradation under solar-light irradiation as measured by a JASCO 750 UV-Vis spectrophotometer at *ca.* 269 nm. It can be observed that the peak obtained for neat phenol (20 ppm) at 269.5 nm gradually decreased in the presence of the as-prepared photocatalysts. Preliminarily, neat NFO and P–CN were tested for phenol degradation and were found to degrade 30% and 68% of the phenol, respectively, under visible light irradiation. But as observed from the graph, the degradation activity of the NFO@P–CN nanocomposite was tremendously enhanced in comparison to neat P–CN and NFO. An excellent activity was observed for the 20 wt% NFO@P–CN, *i.e.*, 96%, which was almost 2-fold higher than P–CN and 3-fold than NFO. It can be observed from Fig. 11b that the photocatalytic activity of the nanocomposite is enhanced with increasing the NFO content up to 20 wt%, while a further increase of the NFO content decreases the photocatalytic performance. This might be due to the increased content of NFO, which might have adsorbed on the active sites on the facades of the photocatalyst, which then hinders the solar light from reaching the plane of the photocatalyst. Therefore, here, the content of NFO in the NFO@P–CN nanocomposite played an important role toward photocatalysis.

### 5.2 Kinetics of phenol oxidation

**Fig. 12:**
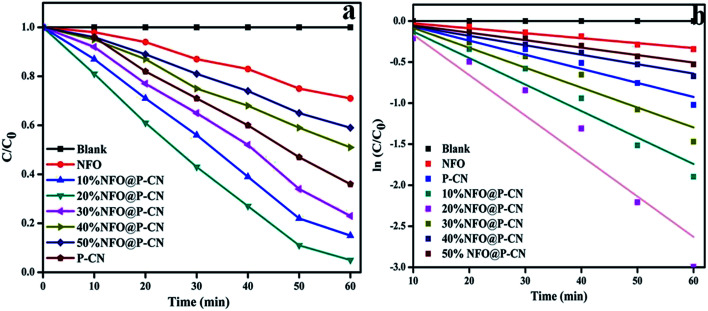
(a) Kinetics study of phenol degradation by the photocatalysts in successive time interval in the first hour of study, (b) kinetics of phenol decolorization in the first cycle.

The kinetics of phenol oxidation of all the as-prepared photocatalysts was studied and analyzed using a UV-Vis spectrophotometer (Fig. 12a–b). The kinetics study was carried out by using 0.02 g of photocatalyst in 20 mL of 20 ppm phenol at pH 6. To assess the kinetics of phenol degradation, the experimental data and the fitting results were analyzed using the zero-order (eqn (7)), first-order (eqn (8)), and second-order models (eqn (9)). From the plotted graphs, the rate constants (*k*), coefficient of determinations (*R*^2^), and average relative errors were evaluated.

7 *C*_t_ = −*k*_0_*t* + *C*_0_

8 *C*_t_/*C*_0_ = e^−*k*_1_*t*^

9 1/*C*_t_ = 1/*C*_0_ + *k*_2_ × *t*

As shown in Fig. 12a, the rate of degradation was determined by using the equation:

Photo decolorization rate = (*C*_0_ − *C*/*C*_0_) × 100. Then, a graph was plotted between concentrations *vs.* time. The reaction was best fitted with the 1st-order rate equation *C*_t_/*C*_0_ = e^−*k*_1_*t*^, where *C*_0_, *t*, and *C*_t_ refer to the initial concentration of the solution, the reaction time, and the solution concentration at reaction time ‘*t*’, respectively. The time-relevant concentration loss of phenol over the NFO@P–CN composite with distinct percentages of NFO under visible light irradiation for 60 min is shown in Fig. 12a. The observed degradation rate constants *k*_obs_ were obtained for the first-order kinetics from Fig. 12b. The rate constants in the 1st-order kinetics of 20 wt% NFO@P–CN was found to be 0.0492 min^−1^, which was almost 8 and 3 times higher than that of the NFO (0.0060 min^−1^) and P–CN (0.0171 min^−1^), respectively. Un summary, from the above study, it was concluded that the phenol degradation over NFO@P–CN followed a 1st-order kinetics.

### 5.3 Effect of scavengers

**Fig. 13:**
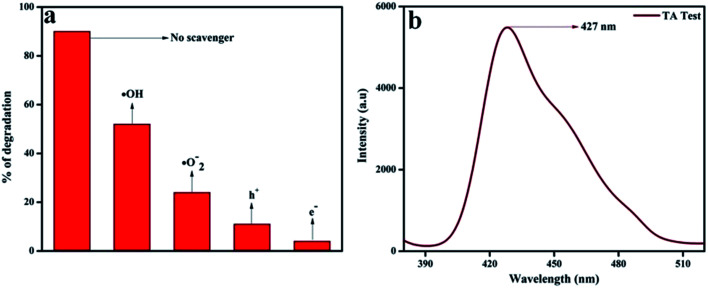
(a) Percentage degradation of phenol over 20 wt% NFO@P–CN in the presence of scavenging agents, (b) confirmation of ˙OH generation in 20 wt% NFO@P–CN through the TA test.

**Scheme 3:**
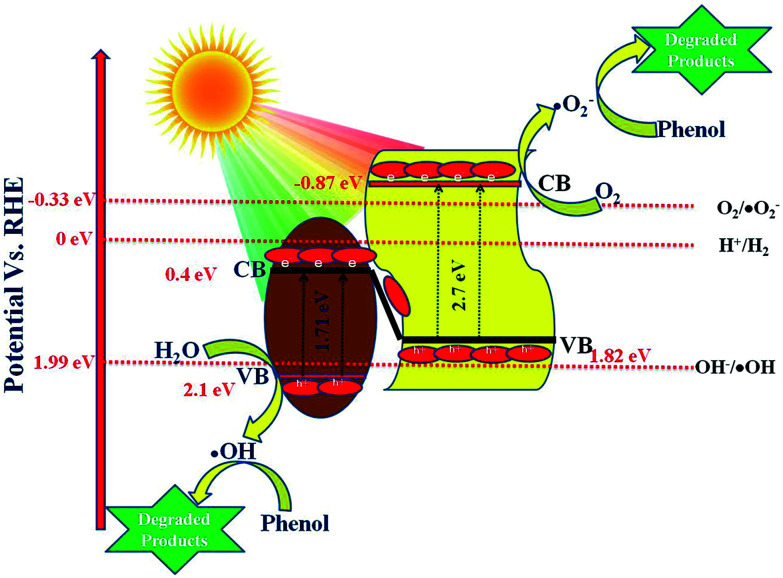
Schematic presentation of the degradation of phenol and evolution of H_2_ over 20 wt% NFO@P–CN.

In order to confirm the major active species responsible for the degradation of phenol, we performed trapping experiments using isopropanol, 1,4-benzoquinone (BQ), citric acid, and DMSO to arrest ˙OH (hydroxyl radical), ˙O_2_^−^ (superoxide radical), h^+^ (holes), and e^−^ (electrons), respectively. In Fig. 13a, it is clearly revealed that the addition of 5 mmol isopropanol suppressed the rate of phenol degradation, whereas the presence of 5 mmol BQ and citric acid showed comparatively better phenol degradation. Finally a noticeable degradation in the case of DMSO was observed. From these observations, it was revealed that the main active species should be ˙OH rather than ˙O_2_^−^ and h^+^, which is in harmony with the hypothesis of the photocatalytic mechanism diagram given in Scheme 3. Again the terephthalic acid (TA) test for 20 wt% NFO@P–CN also confirmed the presence of ˙OH radical by showing a peak at 427 nm (Fig. 13b).

### 5.4 Reusability and stability study

**Fig. 14:**
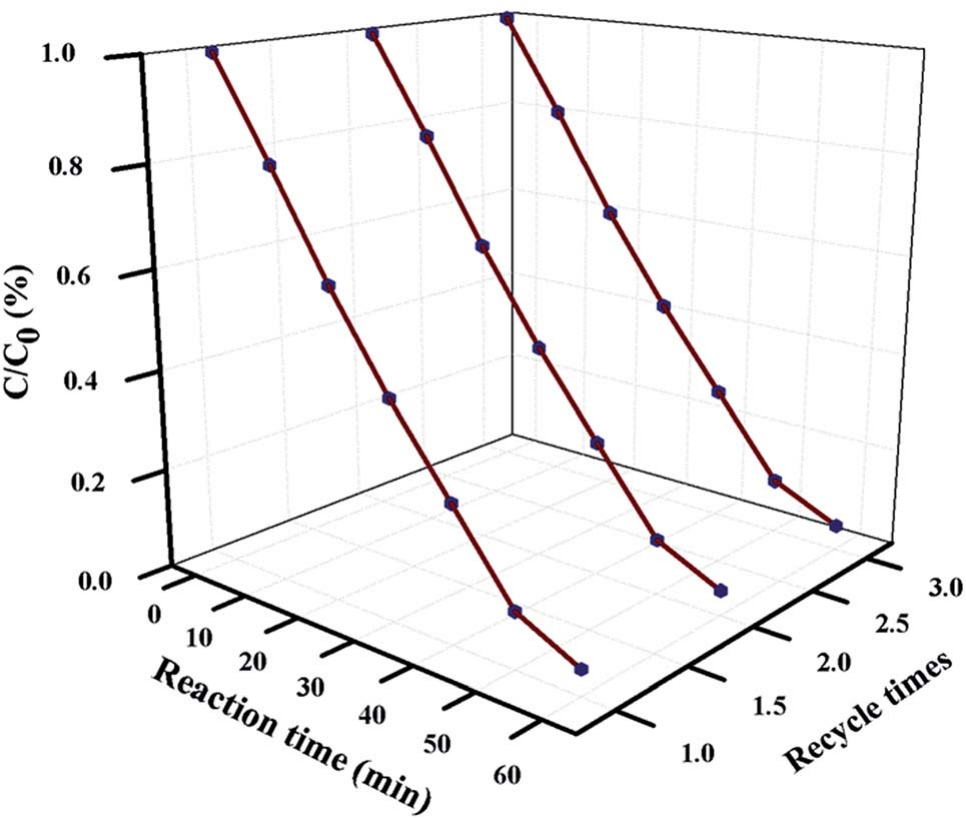
Reusability plot of phenol degradation over 20 wt% NFO@P–CN for 3 consecutive degradation experiments.

For industrial-scale application, it is necessary to test the reusability and stability of 20 wt% NFO@P–CN toward the degradation of phenol. The nanocomposite proved to be photostable for 3 consecutive cycle of degradation experiments. Prior to each degradation experiment, the nanocomposite was washed thoroughly with water and ethanol, followed by drying in an air oven. The catalyst was proved to stable for up to 3 cycles and then its photocatalytic performance decreased, as shown in Fig. 14.

### 5.5 Photocatalytic activity toward the decolorization of the colored organic compound Rhodamine B (RhB) under solar light irradiation

**Fig. 15:**
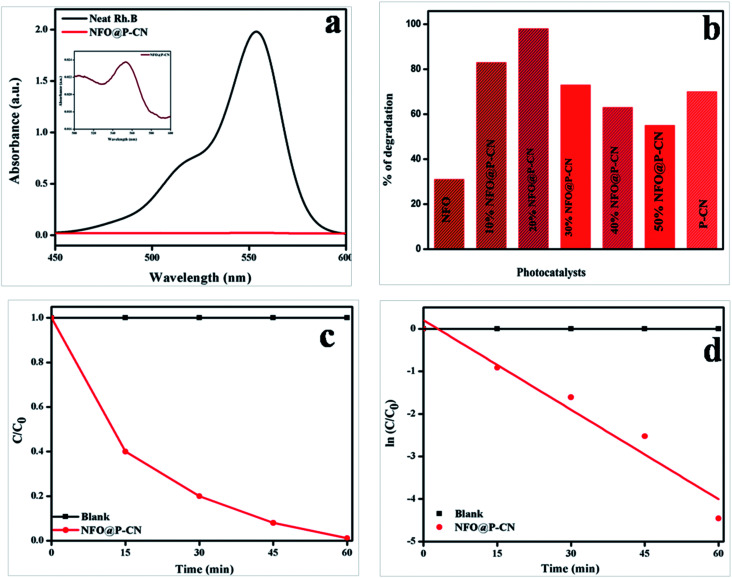
(a) Spectral changes during the decolorization of 10 ppm RhB over 20 wt% NFO@PCN, (b) histogram showing the rate of decolorization by the various prepared catalysts, (c) change of concentration gradient of RhB with respect to the reaction time, (d) kinetics followed by the decolorization process.

The photocatalytic activity of 20 wt% NFO@PCN was tested toward the decolorization of 10 ppm RhB solution under solar-light irradiation. The degradation procedure of RhB was similar to that of phenol. Initially in the absence of catalyst, the RhB solution was decolorized for 1 h under solar-light irradiation and 2% self-decolorization was observed. This was followed by 1 h adsorption mechanism of photocatalyst on active sites of the dye in the dark conditions. There was only an 8% decolorization of RhB solution due to adsorption. In the third step, 0.02 g of the prepared catalysts was used to decolorize 20 mL of 10 ppm RhB solution. The spectral changes in the concentration of the RhB solution by 20 wt% NFO@PCN after decolorization are given in Fig. 15a. From the analysis and from Fig. 15a and b, significant decolorization of about 98.8% was observed for 20 wt% NFO@PCN in comparison to the other prepared composites. It was observed that the percentage of decolorization rate increases with increasing the content up to 20 wt% NFO@PCN. Then, with a further increase in wt%, there is a decrease in the decolorization rate due to blocking of the active sites of the P–CN surface. The order of decolorization of the prepared composites was found to be 20 wt% NFO@PCN > 10% wt% NFO@PCN > 30% NFO@PCN > neat PCN > 40 wt% NFO@PCN > 50 wt% NFO@PCN > neat NFO, as shown in Fig. 15b. With an increase in the time interval of 15 min, the concentration was studied and the color was found to initially be pink to very light pink. The change of concentration with respect to time is plotted in Fig. 15c and the kinetics for the decolorization process was observed to be first order, as shown in Fig. 15d.

### 5.6 Proposed Z-scheme mechanism in 20 wt% NFO@P–CN

As per the band edge positions for NFO and P–CN obtained from MS and the Tauc plots, two types of charge-transfer mechanism can be predicted: a type-II charge-transfer mechanism and a Z-scheme type of mechanism. In the case of the type-II charge-transfer mechanism, based on the band edge positions, the flow of electrons will be from the CB of P–CN to that of NFO and the flow of holes will be from the VB of NFO to the VB of P–CN. If this type of mechanism would exist there would be no generation of H_2_ and ˙OH in the nanocomposite as per the CB of NFO (0.4 eV) and VB of P–CN (1.83 eV), respectively. However in practice, the 20 wt% NFO@P–CN was able to generate 90.4 μmol h^−1^ of H_2_ under visible-light irradiation. In addition, the generation of ˙OH was already confirmed from the TA test. Hence, it can be concluded here that this type of type-II charge-transfer mechanism does not exist in the 20 wt% NFO@P–CN. So, ultimately a Z-scheme type of charge-transfer mechanism should exist in this case. The entire Z-scheme mechanism is schematically presented in Scheme 3. As the scheme represents the photoexcited electrons go from the VB of NFO and its CB and find their way to the VB of P–CN as electrons flow from more negative to less negative or less positive to more positive potential. In the next step, the accumulated electrons at the VB of P–CN get photoexcited to the CB of P–CN. Due to this Z-scheme type of charge-transfer mechanism, a final charge separated state is established, where the assembled electrons at the CB of P–CN capture O_2_ to generate ˙O_2_^−^ as the potential for O_2_/˙O_2_^−^ is −0.33 eV *vs.* NHE. Also, these electrons could be able to generate H_2_ as the CB of P–CN is situated at −0.87 eV. On the other side, the holes that are left behind at the VB of NFO (2.1 eV) form ˙OH as the potential for its generation is 1.99 eV (OH/˙OH = 1.99 eV). All these statements are consistent with H_2_ production and the generation of ˙OH (major active species) in phenol degradation over 20 wt% NFO@P–CN.

10 P–CN + *hν* → P–CN (e^−^ + h^+^)

11 NFO + *hν* → NFO (e^−^ + h^+^)

12 NFO@P–CN (e^−^ + h^+^) → NFO (h^+^) + P–CN (e^−^)

13 P–CN (e^−^) + O_2_ → P–CN + ˙O_2_^−^

14 NFO (h^+^) + H_2_O → H^+^ + ˙OH

15 ˙O^−^_2_ + phenol → degraded products (minor)

16 ˙OH + phenol → degraded products (major)

17 h^+^ + phenol → degraded products (minor)

### 5.7 Proposed photocatalytic hydrogen-evolution mechanism

To make the reduction of H_2_O feasible, the CB edge of a photocatalyst must possess more negative energy with respect to the reduction potential of hydrogen. By analyzing the data, it could be concluded that P–CN has a proper VB and CB positions for both H_2_ and O_2_ generation, but for NFO, the CB position is not appropriate for H_2_ evolution. Again it is noted that, though the CB position of NFO is not suitable for H_2_ evolution, the nanocomposite formed between NFO and P–CN could be able to produce hydrogen. The detailed mechanism of the H_2_ evolution is discussed here.

**Fig. 16:**
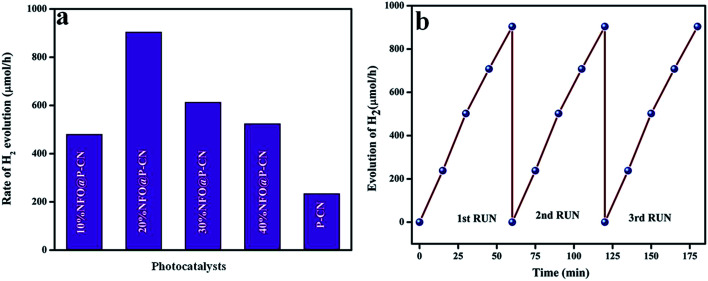
(a) Rate of H_2_ evolution shown by various prepared catalysts, (b) run graph showing the photostability towards H_2_ evolution.

The above reactions indicates that the adsorbed water molecules on the composite particles accept the electrons from the visible-light-induced photocatalytic redox centers on the composites surfaces, causing the reduction of water to H_2_ in the conduction band. Again, due to the good separation of photoinduced charges and superior electrical conductivity of the NFO/P–CN nanomaterial, it was found to be more active in water oxidation. Under the effect of visible-light irradiation (*λ* ≥ 400 nm), all the prepared composites were tested for hydrogen evolution. The data obtained are plotted in Fig. 16a. Before conducting the proposed photocatalytic experiment, a reference experiment was performed under the same ambient conditions by taking a pure triethanolamine solution without using any irradiation or photocatalyst. It was observed that there was no evolution of hydrogen gas. The experiments were performed by taking 20 mL of 20 vol% triethanolamine solution as a sacrificial hole scavenger and taking 0.02 g of the powdered photocatalyst.

The effects of NFO on P–CN toward H_2_ evolution are presented in Fig. 16a and b. NFO is inactive toward H_2_ evolution and it was observed that P–CN was less active for hydrogen production (234 μmol h^−1^) under visible-light irradiation in comparison to the prepared 20 wt% NFO@P–CN composite. Among all the prepared composite photocatalysts, 20 wt% NFO@P–CN evolved the highest amount of hydrogen gas (904 μmol h^−1^) under visible-light irradiation, whereas 10 wt% NFO@P–CN, 30 wt% NFO@P–CN, and 40 wt% NFO@P–CN gave 480, 613, and 524 μmol h^−1^ of hydrogen gas, respectively, under the influence of visible-light irradiation. The 20 wt% NFO@P–CN nanocomposite was found to be a more competent photocatalyst when compared to the other reported photocatalysts. A comparison study is given in Table 1 below. During the H_2_-evolution study, it was observed that there was an increase in the hydrogen-production rate by increasing the NFO percentage up to 20 wt%. Then again, on increasing the quantity of the NFO, the H_2_-production rate significantly decreased. This could be due to the fact that excess NFO might have occupied the active sites on the surface of the photocatalyst and hindered the incident light from getting on to the surface of the photocatalyst. Hence, there was a decrease in electron–hole pair production due to the blocking of the irradiation of light, thus lowering the rate of H_2_ production. Hence from the above discussion, it can be concluded that the NFO@P–CN nanocomposites are able to generate H_2_ at the CB of P–CN only because of the existence of a Z-scheme mechanism in it.

**Table 1:** A comparison study of 20 wt% NFO@P–CN with some other reported photocatalysts

Sample	Light source used	H_2_ μmol h^−1^	Reference
NiFe_2_O_4_	High-pressure Xe lamp (300 W)	0.052	15
g-C_3_N_4_	300 W Xe lamp for 1 h	∼8	36
P–C_3_N_4_	300 W Xe lamp for 1 h	∼24	36
Au@g-C_3_N_4_	300 W Xe lamp for 1 h	∼53	36
g-C_3_N_4_BS (boron, sulphur)	150 W Xe lamp (≥420 nm)	∼53.2	37
Au/g-C_3_N_4_BS	150 W Xe lamp (≥420 nm)	∼94	36
1 wt% Au/g-C_3_N_4_/NiFe_2_O_4_	High-pressure Xe lamp (300 W)	∼16.07	15
20 wt% NFO@P–CN	Medium-pressure 150 W Xe arc lamp (*λ* > 420 nm)	∼904	Prepared photocatalyst

## 6. Reusability

The same experimental protocol was used to test the photostability and reusability of the 20 wt% NFO@P–CN toward H_2_ evolution as followed in phenol degradation. The catalyst was found to be photostable for up to 3 cycles of water reduction reaction.

## 7. Conclusion

In our work, under a systematic methodology, NFO@P–CN nanocomposites were synthesized using a sol–gel and calcination route. The efficient NFO@P–CN nanocomposite designed by doping phosphorus on CN and coupling with NFO was proven to be a promising photocatalyst. A good formation of globular and flake-like P–CN attached with the granular NFO surface was observed from the SEM images. The degradation of phenol followed a first-order kinetics and was found to be 96%, which was almost 2-fold higher than P–CN and 3-fold higher than NFO. A higher amount of H_2_ gas of almost 904 μmol h^−1^ under visible-light irradiation was furnished by 20 wt% NFO@P–CN. The best photocatalytic activity of 20 wt% NFO@P–CN was in agreement with the large visible-light absorption of NFO, optimal NFO loading, and good formation of the synergistic composite. The mechanistic pathway and delay in the recombination of charge carriers were verified from the Z-scheme mechanism, oxygen vacancies, and the electrostatic binding interaction. These results manifest that engineering the surface structure of mixed transition-metal oxides represents a simple and efficient method for constructing high-performance and durable photocatalysts.

## Conflicts of interest

## Supplementary Material
